# Impact of fasting blood glucose on prognosis after acute large vessel occlusion reperfusion: results from a multicenter analysis

**DOI:** 10.3389/fneur.2024.1422851

**Published:** 2024-10-23

**Authors:** Bin Luo, Yi Xiang, Fanlei Meng, Yubo Wang, Zhenzhong Zhang, Hecheng Ren, Lin Ma

**Affiliations:** ^1^Academy of Medical Engineering and Translational Medicine, Tianjin University, Tianjin, China; ^2^Tianjin Neurological Institute, Tianjin Huanhu Hospital, Tianjin, China; ^3^Department of Clinical Laboratory, Tianjin Huanhu Hospital, Tianjin, China; ^4^Department of Neurology, Xianyang Central Hospital, Xianyang, China; ^5^Department of Neurology, Second Hospital of Tianjin Medical University, Tianjin, China; ^6^Department of Neurosurgery, Hengshui Fifth People’s Hospital, Hengshui, China

**Keywords:** acute large vessel occlusion, blood glucose, multivariable logistic regression analysis, mechanical thrombectomy, prognosis

## Abstract

**Objective:**

To analyze the effect of fasting blood glucose levels after reperfusion of acute large vessel occlusion (ALVO) on patient functional prognosis.

**Methods:**

Retrospectively included ALVO patients from three large stroke centers in China, all of whom achieved vascular reperfusion after mechanical thrombectomy or bridging thrombolysis. The prognosis scores of all patients at 90 ± 7 days post-recanalization were categorized into a good prognosis group (mRS 0–2) and a poor prognosis group (mRS 3–6). The relationship between mean blood glucose levels at 72 h post-recanalization and prognosis was explored using multivariable logistic regression analysis. Then we measured the area under the ROC curve for all factors to assess their predictive performance.

**Results:**

(1) Totally 2,056 patients were included in the study, with 1,488 males and 568 females. There were 1,370 patients in the good prognosis group (mRS 0–2) and 686 in the poor prognosis group (mRS 3–6). (2) The two groups exhibited significant differences in terms of age, preoperative mRS score, history of diabetes, and mean fasting blood glucose (MFBG) (*p* < 0.001). (3) With 90-day mRS as the outcome variable, all independent variables were included in Univariate and multivariate regression analyses analysis, and the results showed that: age, preoperative mRS score, history of diabetes, and MFBG are all independent predictors of prognosis after recanalization of ALVO, with MFBG demonstrating a higher predictive power than the other factors (AUC = 0.644).

**Conclusion:**

Various factors are correlated with the prognosis in patients who have undergone ALVO recanalization. Notably, the MFBG level demonstrates a significant predictive value for outcomes within the first 72 h following the recanalization procedure.

## 1 Background

Although mechanical thrombectomy (MT) has achieved higher rates of vascular recanalization in patients with acute large vessel occlusion (ALVO), the issue of reperfusion injury to brain tissue after vascular recanalization has always been a challenge that scholars and clinicians cannot avoid. Approximately 20–50% of ALVO patients may experience stress-induced hyperglycemia, caused by the release of cortisol and adrenaline leading to elevated blood glucose levels, a phenomenon commonly seen even in non-diabetic patients (1, 2). Patients undergoing MT procedures need to consider factors such as surgery-related stress, and hyperglycemia has been associated with adverse clinical outcomes in ALVO patients (3–7). Hyperglycemia levels can further exacerbate the hypoxic status of midbrain cells in the ischemic penumbra, leading to increased acidosis, mitochondrial dysfunction, and even failure (8). Besides, elevated blood glucose levels are associated with the formation of free radicals and activation of matrix metalloproteinases, which can further worsen brain edema (9–11). The 2018 American Heart Association/American Stroke Association (AHA/ASA) guidelines recommend (12) targeting blood glucose levels to 140–180 mg/dL (7.8–10.0 mmol/L), but this standard lacks a higher level of objective evidence due to limited supporting data (1).

Our team’s previous research findings showed: hyperglycemia is an independent risk factor for poor prognosis in ALVO patients after vascular recanalization. The good prognosis rate in the low blood glucose group was 1.62 times higher than that in the hyperglycemia group; and for every 1 mmol/L decrease in blood glucose, the rate of poor prognosis decreased by 7.2% [OR: 0.928, 95% CI (0.879, 0.979), *p* = 0.007] (2). Other studies have shown the opposite: However, other studies have yielded conflicting results, with no significant difference in the 90-day modified Rankin Scale (90ds-mRS) between patients on intensified glucose lowering (4.44–7.22 mmol/L) and patients at standard glucose levels (4.44–9.93 mmol/L) (3–5). Further research is needed to determine whether different blood glucose levels affect the 90ds-mRS in patients.

Taking into account the above factors, we conducted a retrospective study on the fasting blood glucose levels of 2,056 post-MT patients with ALVO from three different medical centers, analyzing the relationship between MFBG levels and 90ds-mRS.

## 2 Materials and methods

### 2.1 Research objects

Data were obtained from three large medical centers in China (Huanhu Hospital in Tianjin, Xianyang Central Hospital, and Tianjin Medical University Second Hospital). These centers had consistent standards for the assessment and treatment of ALVO. We retrospectively collected data from 2,056 patients with acute large vessel occlusion who were seen from January 2019 to June 2023, including 1,488 males (72.4%) and 568 females (27.6%). The number of patients included from Huanhu Hospital in Tianjin was 1,376, and from Xianyang Central Hospital and Tianjin Medical University Second Hospital were 253 and 427, respectively.

#### 2.1.1 Inclusion criteria

① Patients aged 18 or older with acute occlusion of large vessels in the anterior circulation of the brain; ② patients with acute onset, seen within 24 h of normal appearance, diagnosed with AIS by MRI, and confirmed to have ALVO by MRA or DSA; ③ NIHSS score on admission ≥6; ④ pre-stroke mRS ≤1; ⑤ patients with acute occlusion of large vessels in the anterior circulation, meeting the inclusion criteria of the DAWN and DEFUSE-3 trials, occurring between 6–24 h; ⑥ successful recanalization (mTICI ≥2b) was achieved in patients receiving MT or bridging MT after intravenous thrombolysis.

#### 2.1.2 Exclusion criteria

① Incomplete data; ② recurrent stroke; ③ non-occlusion of large vessels in the anterior circulation; ④ patients with post-stroke seizures or those with confirmed patency of occluded vessels partially or completely before MT surgery; ⑤ patients with active bleeding or a tendency to bleed in the perioperative period, coagulation disorders, etc.; ⑥ patients in the time window for intravenous thrombolysis, meeting the indications for intravenous thrombolysis and achieving patency with thrombolytic drugs; ⑦ conditions caused by non-vascular diseases such as tumors, trauma, hematologic diseases, etc.; ⑧ systolic blood pressure ≥185 mmHg or diastolic blood pressure ≥110 mmHg on admission; blood glucose ≤2.7 mmol/L or ≥22.2 mmol/L; ⑨ patients with heart, liver, or kidney dysfunction, unstable vital signs in the perioperative period.

### 2.2 Research method

Research methods according to the latest guidelines from the American Heart Association/American Stroke Association (AHA/ASA) in 2019 (6), screening for intracranial large vessel occlusion was conducted in patients diagnosed with acute stage cerebral infarction. Patients who experienced onset within 4.5 h and met the criteria for intravenous thrombolysis received treatment with recombinant tissue plasminogen activator (rt-PA) at a dose of 0.9 mg/kg. Patients with concurrent acute large vessel occlusion underwent mechanical thrombectomy in conjunction with intravenous thrombolysis. Patients with a high thrombus burden underwent direct stent retriever thrombectomy or a combination of stent retriever thrombectomy and aspiration therapy. For patients with stenosis or dissection, angioplasty was performed in conjunction with mechanical thrombectomy, and for patients with large arterial occlusion related to atherosclerosis, antiplatelet aggregation drugs and statins were used intraoperatively. The modified Thrombolysis in Cerebral Infarction classification (mTICI) was used to evaluate the effectiveness of vascular recanalization, with perfusion reaching mTICI 2b or 3 considered a successful recanalization. In case that fasting blood glucose levels were outside the range of 3.9–6.1 mmol/L or postprandial blood glucose exceeded 11.1 mmol/L, medical intervention was provided immediately to stabilize the patient’s blood glucose levels between 3.9–7.8 mmol/L (6, 7).

#### 2.2.1 Extraction of blood glucose data

Blood glucose values were collected using portable glucose meters, obtaining nine fasting blood glucose for each patient within 72 h post-surgery (for three consecutive days, three times a day with a 30-min interval between each measurement), and calculating the mean fasting blood glucose level (MFBG).

#### 2.2.2 Outcome assessment measure

The 90-day modified Rankin Scale (90ds-mRS) (8) is used to evaluate the degree of functional impairment in stroke patients. A good prognosis was defined as 90ds-mRS (0–2), and a poor prognosis was defined as 90ds-mRS (3–6). The mRS scale ranges from 0–6, with 0 indicating no residual symptoms, and 6 indicating death. 0 point: no residual symptoms; 1 point: residual symptoms present, but no functional impairment, able to live normally; 2 points: mild residual disability, but still able to live independently; 3 points: moderate disability, able to walk independently but requires assistance with some daily activities; 4 points: moderate to severe disability, unable to walk independently, requiring assistance with most daily activities; 5 points: severe disability, bedridden, incontinence, completely dependent on others for daily activities; 6 points: death.

### 2.3 Statistical analysis

All statistical analyses were accomplished via SPSS version 27.0. Categorical variables were represented as frequencies in conjunction with corresponding percentages (*n*%), and inter-group comparisons were appraised by the chi-square test (*χ*^2^) or Fisher’s exact test, as deemed appropriate. Continuous variables were delineated using median values and interquartile ranges [median (IQR)], and inter-group dissimilarities were inspected through the student’s *t*-test. Subsequently, univariate logistic regression analysis was implemented to identify potential predictors of the 90-day modified Rankin Scale (90ds-mRS) outcome. Variables having a *p*-value less than 0.1 were thereafter integrated into a multivariate logistic regression model to formulate a predictive equation. A *p*-value less than 0.05 was considered statistically significant.

## 3 Results

**Figure 1:**
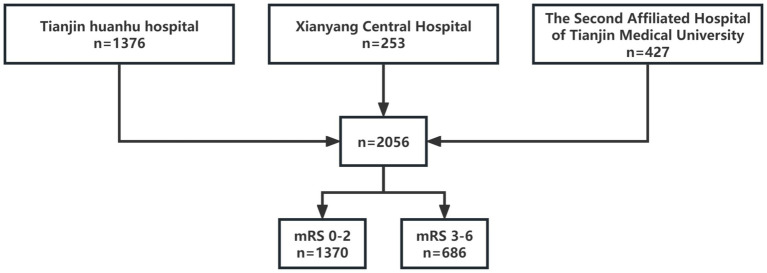
Number of samples enrolled per center and samples stratified by 90-day mRS.

A total of 2,056 patients with acute large vessel occlusion were enrolled in this study, with an average age of 63 (55, 71) years. There were 1,488 males (72.4%) with a mean age of 64 (57, 71) years and 568 females (27.6%) with a mean age of 67 (60, 74) years. In the study population, a total of 1,370 patients were classified into the good outcome group (mRS 0–2), while 686 patients were categorized into the poor outcome group (mRS 3–6) (Figure 1).

### 3.1 Baseline characteristics were compared between the two groups

Following recanalization, an analysis of the mRS 0–2 group versus the mRS 3–6 group revealed significant intergroup differences across multiple factors, with the exception of gender, which exhibited no distinction between the two groups. Notably, the median age was found to be higher in the group with poor outcomes (63 years vs. years). The proportion of patients with a preoperative mRS score of 3–6 who experienced poor outcomes increased post-recanalization (96.1% vs. 90.9%), while the rate of favorable outcomes for those with a preoperative mRS score of 0–2 decreased (from 9.1 to 3.9%). This pattern was also evident in the subgroup analysis of patients with and without a history of diabetes: the rate of positive outcomes declined among patients without a history of diabetes (from 80.2 to 61.2%), and the rate of negative outcomes rose among patients with a history of diabetes (from 19.8 to 38.8%) (Table 1).

**Table 1:** The baseline data of all patients were compared.

Variables		90ds-mRS (0–2) *n* = 1,370	90ds-mRS (3–6) *n* = 686	*p*
Gender	Female (*n*%)	366 (26.7%)	202 (29.4%)	0.192
	Male (*n*%)	1,004 (73.3%)	484 (70.6%)	
Age [m (IQR)]		61 (53, 69)	63 (57, 72)	<0.001^*^
Preoperative mRS	0–2 (*n*%)	125 (9.1%)	27 (3.9%)	<0.001^*^
	3–6 (*n*%)	1,245 (90.9%)	659 (96.1%)	
Diabetes mellitus	No (*n*%)	1,099 (80.2%)	420 (61.2%)	<0.001^*^
	Yes (*n*%)	271 (19.8%)	266 (38.8%)	
MFBG (mmol/L) [m (IQR)]		8.7 (7.1, 8.7)	8.7 (7.6, 11.2)	<0.001^*^

^*^p < 0.05. m (IQR), median (interquartile range); 90ds-mRS, modified Rankin Scale at 90 days of onset.

### 3.2 Analysis of risk factors with 90d-mRS as the outcome variable

**Figure 2:**
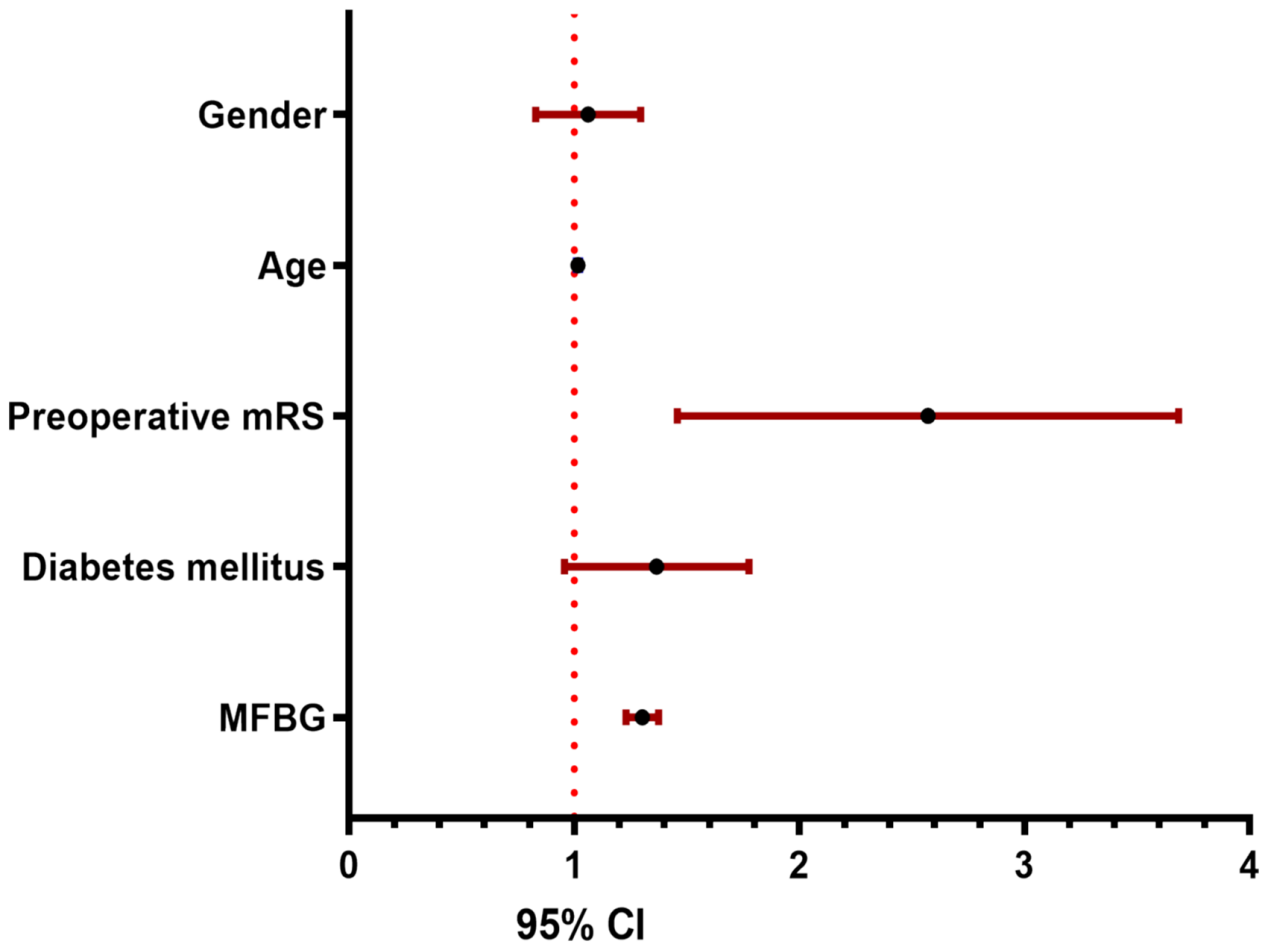
90ds-mRS forest map of risk factors.

Univariate logistic regression analysis was implemented to identify potential predictors of the 90-day modified Rankin Scale (90ds-mRS) outcome. Variables having a *p*-value less than 0.05 were thereafter integrated into a multivariate logistic regression model to formulate a predictive equation. The results demonstrated that age, preoperative mRS, diabetes mellitus, and MFBG are all independent predictors of the 90-day mRS in patients with acute intracranial large vessel occlusion (ALVO) following recanalization. Calculation of the area under the receiver operating characteristic (ROC) curve for these factors revealed that MFBG possesses the highest predictive efficacy, with a value of 0.644 (Table 2 and Figure 2).

**Table 2:** Logistic regression analysis and AUC of ROC.

Variables	Univariate regression		Multivariable logistic regression		AUC
	OR (95% CI)	*p*	OR (95% CI)	*p*	
Gender	0.873 (0.713, 1.070)	<0.001^*^	1.044 (0.837, 1.303)	0.703	0.528
Age	1.020 (1.012, 1.029)	<0.001^*^	1.018 (1.009, 1.027)	<0.001^*^	0.579
Preoperative mRS	2.451 (1.600, 3.754)	<0.001^*^	2.410 (1.547, 3.756)	<0.001^*^	0.501
Diabetes mellitus	2.568 (2.096, 3.147)	<0.001^*^	1.143 (1.117, 1.838)	0.005^*^	0.531
MFBG	1.356 (1.292, 1.423)	<0.001^*^	1.302 (1.231, 1.376)	<0.001^*^	0.644

^*^p < 0.05. OR, odd ratio; CI, confidence interval; mRS, modified Rankin Scale; MFBG, mean fasting blood glucose; ROC, receiver operating characteristic; AUC, area under the curve.

## 4 Discussion

Our study confirmed that blood glucose levels following recanalization of acute large vessel occlusion in the anterior circulation is independently associated with worse 90-day outcomes in patients. This suggests that even with successful vascular recanalization, a patient’s prognosis remains influenced by their blood glucose levels. Indeed, demographic age, preoperative modified Rankin Scale (mRS) score, and the presence of a diabetic comorbidity are pivotal in determining clinical outcomes for patients. Nevertheless, despite these variables, mean fasting blood glucose (MFBG) emerges as a superior predictor of 90-day modified Rankin Scale (90-day mRS) outcomes within the critical 72-h window following achieving successful cerebral reperfusion, with an area under the curve (AUC) of 0.644.

Our study aims to explore the relationship between fasting blood glucose levels within 72 h after recanalization of large vessel occlusion in the anterior circulation and patients’ 90-day outcomes. This differs from other studies, which largely have not discussed whether the occluded vessels were successfully recanalized. Although their findings are consistent with ours, whether non-recanalized vessel occlusion is an unpredictable confounding factor affecting patient outcomes is worth considering. Moreover, many studies have focused only on pre-recanalization blood glucose levels, yet stress-induced hyperglycemia is a common phenomenon (5, 9–11). There are also studies that indicate glycated hemoglobin (HbA1c) is an independent influencing factor on the prognosis of patients with large vessel occlusion; however, HbA1c represents the average blood glucose level over an 8–12 weeks period, but its broad time span and numerous confounding factors make it less than ideal for reflecting the uniform treatment conditions in a hospital setting (11). Therefore, we conducted our study using the mean random blood glucose level within 72 h after vessel recanalization as a potential factor. Research by Yao et al. (12) on the SMART study (*n* = 2,862) demonstrated: the finding that there was a significant association between elevated blood glucose after stroke and poor function in patients with diabetes who were not previously diagnosed confirms the importance of controlling blood glucose levels during the acute phase of AIS. Kim et al. (13) collected data from 309 thrombectomy patients, indicating that for every 1 mmol/L increase in blood glucose levels, the rate of poor prognosis increased by 10.6%. These findings are consistent with our current study as well as with our previous research outcomes: hyperglycemia is an independent risk factor for poor prognosis post-vessel recanalization in ALVO patients; the rate of favorable prognosis in the low blood glucose group is 1.62 times higher than the hyperglycemia group; for every 1 mmol/L increase in blood glucose, the rate of poor prognosis increases by 7.2% [OR: 0.928, 95% CI (0.879, 0.979), *p* = 0.007] (2). Possible cause: hyperglycemia may delay the resolution of focal brain edema in the ischemic brain region, reducing reperfusion blood flow in the ischemic brain tissue. Hyperglycemia can exacerbate intracellular acidosis in the ischemic penumbra cells and worsen brain damage (14). Desilles et al. (15) found that hyperglycemia triggers a cascade of thrombo-inflammatory reactions, amplifying downstream microvascular thrombo-inflammation induced by arterial occlusion in the brain. Hyperglycemia increases oxidative stress and protein hydrolysis at the blood-brain barrier, further leading to brain ischemia-reperfusion injury, brain edema, and even hemorrhage. It suggests that in clinical practice, high attention should be paid to ALVO patients with elevated blood glucose levels, with proper blood glucose control, dietary management, and personalized treatment options being crucial for improving prognosis rates. Although there are various methods for measuring blood glucose, more convenient and economical methods are optimal. Consequently, we used the average fasting blood glucose levels over 72 h from multiple medical centers for our study. Furthermore, we only included data from patients who had successful recanalization, and then we applied the PSM method to match and calibrate the data, which has enhanced the accuracy of our results.

It is inescapable that age serves as a significant determinant of patient prognosis, potentially attributable to the higher National Institutes of Health Stroke Scale (NIHSS) scores at the time of stroke onset in elderly patients, as well as an increased propensity for perioperative complications such as pneumonia, infections, and cardiac emergencies (16–18). Preoperative modified Rankin Scale (mRS) scores are significantly associated with prognosis, as the duration of ischemia, level of consciousness, location, and volume of ischemic infarction all contribute to the preoperative mRS score, which in turn has a determinant role on patient outcomes.

Our study indicates that diabetic patients exhibit a worse 90ds-mRS compared to those without a history of diabetes, which may be attributed to a differential response to acute stress hyperglycemia associated with acute large vessel occlusion (ALVO) stroke between diabetic and non-diabetic individuals. Diabetic patients may exhibit a higher fasting glucose-to-glycated hemoglobin ratio, potentially leading to a distinct subtype of stroke. Moreover, differences in mean blood glucose levels, incidence of hypoglycemia, and variability in glucose levels between diabetic and non-diabetic patients may ultimately influence their clinical outcomes; diabetic individuals may exhibit a blunted response to hypoglycemia, hyperglycemia, and fluctuations in blood glucose levels (19).

## 5 Conclusion

In conclusion, various factors are correlated with the prognosis in patients who have undergone ALVO recanalization. Notably, the MFBG level demonstrates a significant predictive value for outcomes within the first 72 h following the recanalization procedure.

## 6 Deficiencies of this study

1. This study is retrospective in nature.
2. There is a lack of higher-level clinical research conclusions on the relationship between post-reperfusion blood glucose levels in vascular occlusion patients and patient prognosis, necessitating more large-scale, multicenter, randomized controlled trial data for further validation.
3. The paucity of selected variables may result in bias of the research findings. To address this limitation, our impending prospective, standardized enrollment study necessitates the inclusion of a broader array of factors to rectify this deficiency. This augmentation will encompass a more comprehensive suite of laboratory assays and imaging studies, thereby enhancing the robustness and generalizability of the study outcomes.

## Data Availability

The raw data supporting the conclusions of this article will be made available by the authors, without undue reservation.
